# Dogs are not better than humans at detecting coherent motion

**DOI:** 10.1038/s41598-017-11864-z

**Published:** 2017-09-12

**Authors:** Orsolya Kanizsár, Paolo Mongillo, Luca Battaglini, Gianluca Campana, Lieta Marinelli

**Affiliations:** 1 Laboratory of Applied Ethology, Dipartimento di Biomedicina Comparata e Alimentazione, University of Padova, Viale dell’Università 16, 35020 Legnaro, (PD) Italy; 2 Dipartimento di Psicologia Generale, University of Padova, Via Venezia 8, Padova, (PD) Italy

## Abstract

The ability to perceive motion is one of the main properties of the visual system. Sensitivity in detecting coherent motion has been thoroughly investigated in humans, where thresholds for motion detection are well below 10% of coherence, i.e. of the proportion of dots coherently moving in the same direction, among a background of randomly moving dots. Equally low thresholds have been found in other species, including monkeys, cats and seals. Given the lack of data from the domestic dog, we tested 5 adult dogs on a conditioned discrimination task with random dot displays. In addition, five adult humans were tested in the same condition for comparative purposes. The mean threshold for motion detection in our dogs was 42% of coherence, while that of humans was as low as 5%. Therefore, dogs have a much higher threshold of coherent motion detection than humans, and possibly also than phylogenetically closer species that have been tested in similar experimental conditions. Various factors, including the relative role of global and local motion processing and experience with the experimental stimuli may have contributed to this result. Overall, this finding questions the general claim on dogs’ high performance in detecting motion.

## Introduction

Perceiving motion, as one of the main properties of the visual system, is among the first features of visual abilities that started to develop through evolution^1^. The detection of movement in the environment is crucial for adaptive behaviour, such as recognizing predators and preys. Sensitivity to coherent motion has led to a large body of research in various non-human species, as well as in different populations of humans. The perception of coherent motion starts with the detection and processing of information from several local motion units, enabling the perceptual system to build the representation of speed and direction of global motion^2^. Individuals’ sensitivity in the perception of coherent motion is typically assessed by the use of random-dot displays^3^, visual stimuli composed of a certain number of dots coherently moving in the same direction (signal dots), among dots moving in random directions (noise dots). The lower the proportion of signal dots in the display, the harder it is to discriminate the latter from displays composed only of noise dots. Detection thresholds are defined as the minimum proportion of coherently moving dots that allows a subject to reliably discriminate (with an arbitrarily chosen accuracy, generally set at 75%) the stimulus containing signal dots from a pure noise stimulus. Thus, in experimental procedures the proportion of signal dots is systematically varied, and detection accuracy is used to compute individual psychometric curves and thresholds as a function of the proportion of signal dots.

The lowest thresholds reported for humans are well under 10% of coherence, although some variability exists across studies, possibly due to methodological differences^4, 5^. Similarly, low thresholds have also been reported for several non-human species, including monkeys^3^, cats^6, 7^, and seals^8^. Higher thresholds, in the range of 20% to 60%, are reported for other species, such as pigeons^9^, rats and mice^10^. Higher thresholds are also found in specific human populations, such as children^11–13^, adults with autism^14^ or dyslexia^4, 5^.

Due to their history of domestication and convergent evolution with humans, dogs have faced challenges of adapting to the human environment, which makes them one of the most compelling species to investigate human cognition from a comparative aspect. Accordingly, in the last decades several studies have investigated dogs’ abilities of using visual cues and reported that dogs have a special ability to use visual cues in communicating with humans, involving pointing, looking, bowing^15, 16^, as well as relying on complex and subtle visual cues of emotional facial expressions^17^. Beside these studies on cognitive mechanisms underlying the dogs’ ability to use visual cues, few behavioral studies looked at more basic functions of dogs’ visual system and have revealed that dogs are able to discriminate global and local features of static visual stimuli^18^ and to discriminate biological- from non-biological motion^19^. However, to the best of our knowledge, studies about sensitivity of detecting coherent motion in dogs are lacking.

From a physiological point of view, the fundaments of dogs’ vision have been deeply investigated. Most of the differences in visual perception between dogs and humans have been attributed to structural differences of the retina, and particularly in the number, distribution and neural connections of retinal photoreceptors, rods and cones^20–22^. On the one hand, a lower concentration of cones in the central area of the retina and a higher degree of convergence of these photoreceptors on ganglion cells justifies a visual acuity 4 to 7 times lower in dogs than in humans^21^. Indeed, some findings indicate that such lower acuity is due to the structure of the retina and not to other optical properties of the eyes or post-retinal processing^23^. On the other hand, a higher number of rods, and their more homogeneous distribution, including the *area centralis* of the retina (which completely lack rods in humans), contributes to dogs’ higher sensitivity to light and an advantage over humans to see under dim light conditions. Interestingly, rods are also the photoreceptors primarily implied in the perception of motion; thus, the high number of rods in canine’s retina has been suggested to play a part in dogs claimed high sensitivity towards moving stimuli^21^. However, to the best of our knowledge, the only study investigating dogs’ sensitivity to moving targets dates back to the first half of the 20th century^21^, and no effort has been made in more recent times to replicate those findings, or to further investigate dogs’ ability to detect coherent motion, neither *per se* nor from a comparative standpoint.

On these bases, we aimed to investigate the sensitivity of dogs for detecting coherent motion, using random dot displays in a two-way conditioned discrimination procedure. In addition, for a direct comparison with dogs, we investigated adult humans’ thresholds of perception of coherent motion, in the same experimental conditions (i.e. with stimuli having the same parameters of size, density and speed and a similar assessment protocol) of our dogs.

## Results

### Dogs

The dogs needed between 33 and 85 sessions (median = 44) to reach the criterion of choosing the target stimulus (the one containing signal dots) with at least 90% of success in the training phase; all dogs maintained this success rate throughout the experiment.

**Figure 1:**
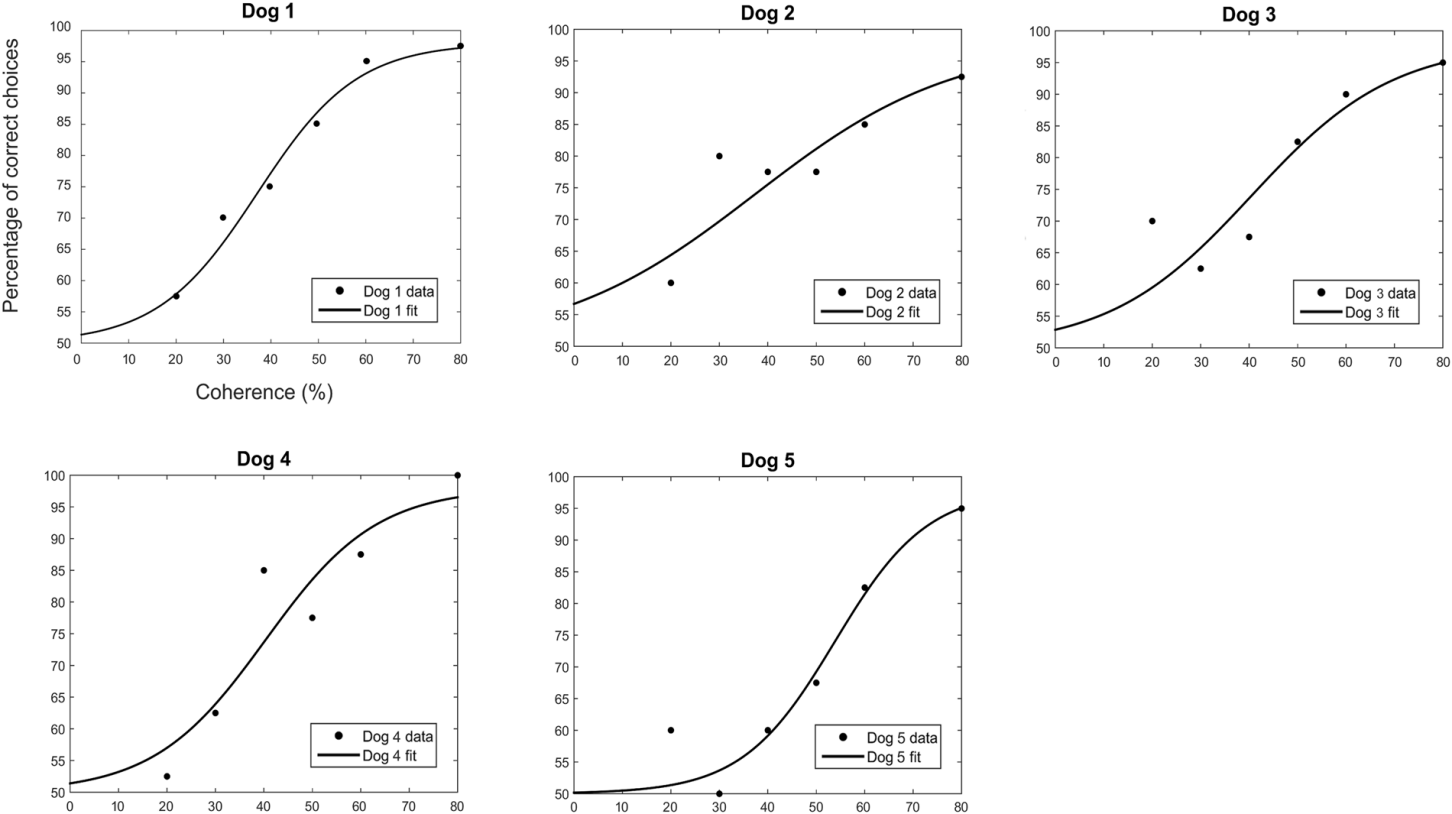
Psychometric curves of dogs as a function of the percentage of coherence. The charts illustrate the psychometric functions (black lines) for each of the five dogs, obtained by fitting the percentage of correct choices (black dots) for each level of coherence.

Figure 1 shows the psychometric functions of each dog and their percentage of correct choices for each level of coherence. Table 1 reports the *Alpha* and *Beta* parameters and their standard deviation for each dog. The mean threshold of coherent motion detection in dogs was at 42.2% of coherence. The mean value of the slope of the dog’s psychometric function was 0.08.

**Table 1:** Values of the *Alpha* and *Beta* parameters and the estimated standard deviation for each of the five dogs.

	*Alpha*	SD *Alpha*	*Beta*	SD *Beta*
Dog 1	37.6	3.7	0.095	0.04
Dog 2	37.4	5.8	0.048	0.01
Dog 3	40.5	4.4	0.068	0.02
Dog 4	41.8	3.9	0.086	0.03
Dog 5	53.9	3.5	0.104	0.07

### Humans

All the participants reached the learning criterion within the minimum amount of six training sessions and they remained above this criterion for all the remaining training trials with 100% of success.

**Figure 2:**
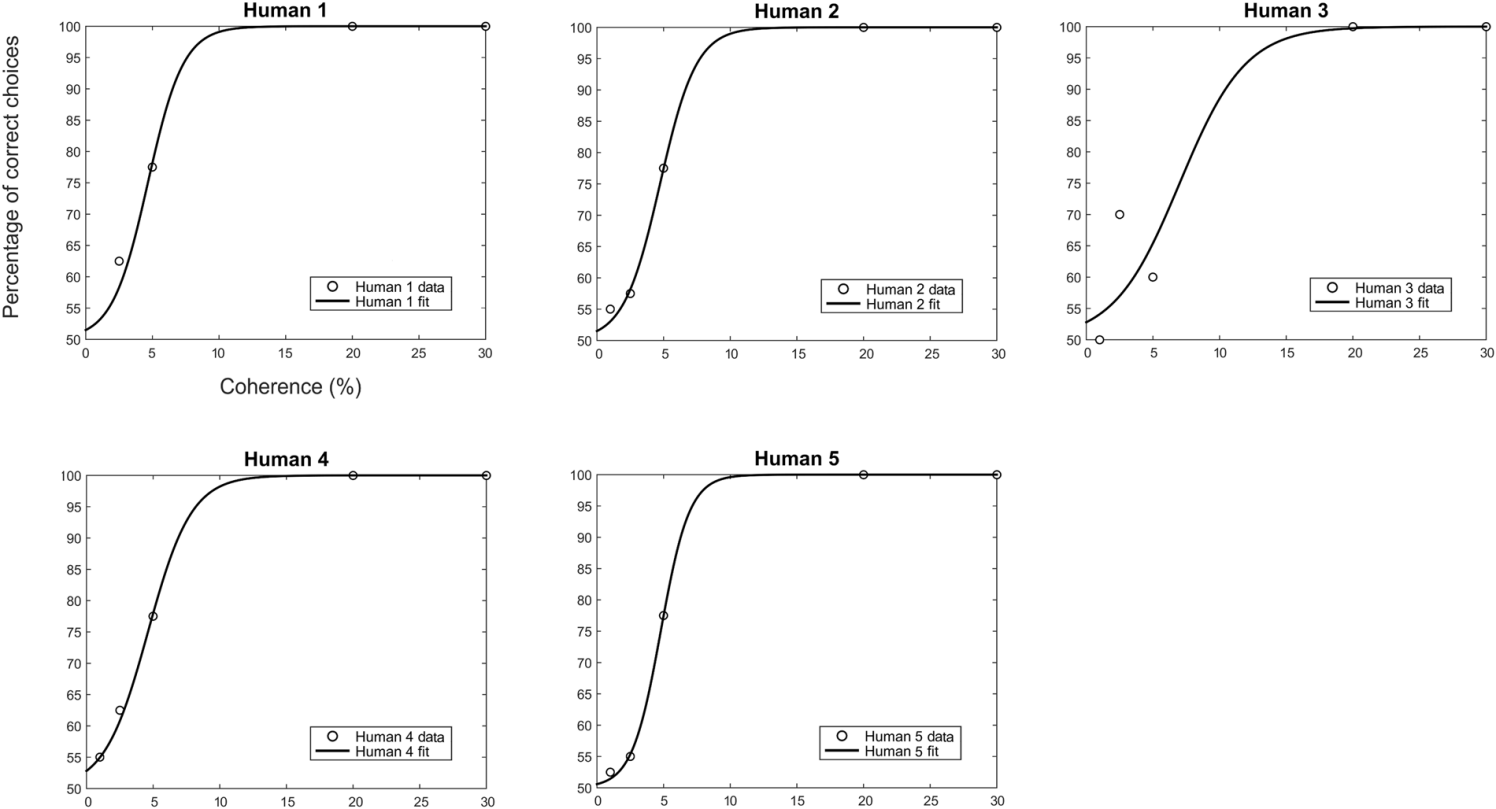
Psychometric curves of humans as a function of the percentage of coherence. The charts illustrate the psychometric functions (black lines) for each of the five humans, obtained by fitting the percentage of correct choices (empty dots) for each level of coherence.

Figure 2 shows the psychometric function of each human participant and their percentage of correct choices for each level of coherence. Table 2 reports the *Alpha* and *Beta* parameters and their standard deviation for each human participant. The mean threshold of coherent motion detection in humans was at 5.1% of coherence. The mean value of the slope of the human participant’s psychometric function was 0.68.

**Table 2:** Values of the *Alpha* and *Beta* parameters and the estimated standard deviation for each of the five humans.

	*Alpha*	SD *Alpha*	*Beta*	SD *Beta*
Human 1	4.6	0.77	0.74	2.2
Human 2	4.7	0.66	0.73	2.3
Human 3	7.0	1.67	0.40	2.1
Human 4	4.6	0.73	0.61	1.5
Human 5	4.7	0.50	0.92	3.1

Humans’ *Alpha* was significantly higher (t = −12.08, P < 0.001) and humans’ *Beta* significantly lower (t = −6.94, P = 0.002) than that of dogs.

## Discussion

In this study, we investigated coherent motion detection thresholds in domestic dogs, i.e. their ability to discriminate a signal stimulus with a variable proportion of dots moving in the same direction, from randomly moving dots display, with an accuracy of 75%. On average, dogs’ threshold was equal to 42.2% coherence level of the signal stimulus. The threshold of human subjects tested in the same condition was significantly lower, with an average value of 5.1%.

This study included an initial training, which was successfully completed by all dogs within 80 training sessions, in line with other studies investigating various aspects of dogs visual processing^18, 24, 25^. None of the dogs had difficulty in maintaining the set criterion in the training presentations of the test session. These findings support this procedure as a viable method for investigating motion processing in domestic dogs.

The range of thresholds found in our dogs spanned between 37% and 54%. Individual data shows that most of our subjects’ thresholds fell in the 40% neighborhood, and only one subject’s threshold seemed to deviate from this value. Nothing in the performance of the latter subject during training and test (e.g. speed of learning, ability to maintain criterion) or in its behavior, suggested explanations for its higher threshold not linked to motion processing, such as a lack in motivation, or learning difficulties. In addition, the overall variability shown by our dogs was proportionally lower than that of our human subjects, or that reported for other species, including pigeons^9^, and cats^6, 7^. Thus, we should retain this range as representative of a physiological individual variability in dogs’ thresholds for coherent motion detection.

Dogs’ threshold was considerably higher than that of our human participants. Of relevance, the consistent experimental condition soothed the impact on results of methodological differences; on the contrary, the latter hinder the possibility of a proper comparison with other studies. Factors such as the characteristics of stimuli, technologies to present stimuli and record data, and the type of populations involved (e.g. captive/experimental vs. companion animals), are source of substantial differences in thresholds for coherence motion detection. For instance, two independent studies report thresholds for cats between 5% and 9% in one case^7^, and around 25% in the second case^6^; a similar across-study variability is found in humans, with reported thresholds for healthy adult individuals ranging from 5% to 25%^9, 26^. Even within the same study, modification of stimulus parameters, such as dot density, lifetime or speed, can dramatically influence detection thresholds in both humans and animals^4, 8, 26^. In this sense, the difference observed between our dogs and humans in the same experimental condition acquires particular significance, as it speaks against claims of a better, or even just a comparable ability of dogs in perceiving coherent motion with respect to humans.

What could be the source of such striking difference? From the neurobiological standpoint, the place to look at would be the cortical areas where the processing of motion is believed to occur; in humans, these processes are centered in the middle temporal area, and its up- and down-stream connections^27^. There are sufficient differences between humans and dogs in the neuroanatomical structure of these neural pathways, to suggest that mechanisms and the limits of motion detection differ between these taxa^28, 29^. A previous study comparing humans and pigeons in the same tasks, reports values of humans’ thresholds very similar to our human participants and pigeons’ thresholds roughly similar to those of our dogs^9^. Pigeons’ lower performance were attributed to a poorer integration of motion signals at both the local level, i.e. integrating the movement of a few dots across relatively long time intervals, and at the global level, i.e. integrating the paths of many dots across a large area of the display. Both mechanisms could have contributed towards the difference in detection of coherent motion by our dogs and humans. Our stimuli featured a relatively long dot lifetime (i.e. 1 s), allowing local motion integration to occur, and a high enough dot density to facilitate sampling of several dots at the same time, thus allowing global integration mechanisms. As such, we cannot speculate on which, if any, of these two mechanisms has more weight in explaining the differences between dogs and human, and further studies are needed to clarify this aspect.

One further aspect that could have contributed to the high threshold found in our dogs is experience with these types of/or with these specific stimuli. Although our dogs received 100 test presentations (20 per coherence level), in addition to a much higher number of training presentations, it is possible that their performance had not yet stabilized at the end of the testing phase. Effects of experience have indeed been documented, e.g. for mice^10^, monkeys^30^ and seals^8^. In the latter, individual threshold decreased from 33.7% to 4.7% across the study. Although concurrent variations in other parameters do not allow a precise estimate of the effects of experience, these findings warrant verifying if our dogs’ thresholds could be improved through further exposition to the experimental stimuli.

Regardless of the underlying mechanisms, comparative aspects of motion detection could also be looked at from an ecological perspective. In this sense, feeding strategies not relying on detecting movement, such as scavenging, predominate in the ecological niche occupied by the so-called village dogs, which are believed to provide a good example of dogs in earlier stages of domestication^31^. Thus, canine domestication may have relaxed pressure on the need for a visual system highly specialized in motion detection.

In conclusion, this study indicates that the threshold for the detection of coherent motion is higher in dogs than it is in humans. What precise mechanisms underlie these differences is still to be investigated. Possible factors include experience, and the relative role of local and global motion processing, which are currently being addressed by our research group.

## Methods

### Subjects

#### Dogs

Our sample was comprised of five pet dogs, three females and two males, between 3 and 11 years of age. The sample included one dog for each of the following breeds: Cocker Spaniel, Golden Retriever, Labrador-Poodle mix (‘Labradoodle’), Mudi, and Siberian Husky. The owners were all workers and students of the University of Padova and participated in the experiments on a voluntary basis. All subjects underwent a veterinary examination before being enrolled in the tests and did not have any health conditions that would prevent them from participation. Dogs were selected according to high motivation for food and the willingness to cooperate and feel comfortable with being in the laboratory.

#### Humans

Our sample comprised five volunteers, three females and two males, between 25 and 45 years of age. Subjects were selected on the criterion that they were not familiar with stimuli and task.

### Stimuli

Stimuli were created with MATLAB (MATLAB version 7.10.0. Natick, Massachusetts: The MathWorks Inc., 2010), using features of Psycho Toolbox^32, 33^. The stimuli were shown on a black squared area of 31.1 × 31.1 cm (24.0 × 24.0 deg, from the viewing distance of 70 cm), where white dots with a diameter of 0.16 cm moved at a speed of 19.4 cm/s (15.0 deg/s). Each dot had a lifespan of 1 s, after which it disappeared and was regenerated in a different part of the display. There was a total of 5000 dots moving in the display, for a density of 5.9 dots/cm^2^ (8.7 dots/deg^2^). Dot size, density and speed were chosen based on stimuli that were previously used for testing other species in similar experiments^8, 34^, and, for dot size, also on known physiological values of visual acuity in dogs^21^. For the training phase, the target stimulus was set at a coherence of 80%, i.e. 80% of the dots moved in the same direction (towards the left side of the display), whereas the remaining 20% moved in random directions. In the test phase (see below), subjects were presented with a set of target stimuli with five levels of coherence (varied within blocks). For dogs these were 60%, 50%, 40%, 30% and 20%; for humans they were 30%, 20%, 5%, 2.5% and 1%. The levels of coherence for the test stimuli were created in accordance with previous studies in both human and non-human species^8, 9, 34^. The non-target stimulus had a coherence level of 0%, that is all of the dots moved in random directions, in all trials of the training and test phase.

### Experimental setting

All the experiments took place in the Laboratory of Applied Ethology of the Department Biomedicine and Food Science (University of Padova), in a testing area of 2.5 × 3 m. Stimuli were presented on two identical monitors (VG248QE, ASUSTeK Computer Inc., Taipei, Taiwan), whose refresh rate was set at 120 Hz; this setting was meant to prevent possible biases on dogs’ detection of motion, due to their higher flicker fusion frequency^21^. Monitors had touch-screen capabilities, so touches of their surface (i.e. choices of either stimulus, as detailed below) were automatically recorded. Monitors were connected to a PC (Optiplex 960, Dell Inc., Round Rock, Texas, USA). Monitors were placed 25 cm away from each other, on two height-adjustable stands, so their height could be set at eye level for each subject. Presentations were controlled with a Bluetooth keyboard (Logitech K400R, Logitech International S.A. Losanna, Switzerland).

### Procedure for dogs

#### General trial procedure

Initially, dogs underwent a preliminary phase, in which they were shaped to touch the screen with the nose and got accustomed to the trial procedure. During each trial, subjects were standing or sitting beside the experimenter (O.K.) who held the dog gently by its harness, within a marked area at 75 cm from the monitors. When the dog was oriented toward the monitors, the experimenter closed her eyes to avoid influencing the subjects’ choice and started the presentation of the stimuli. The non-target and the target stimuli appeared, one on each monitor, and remained visible until subject’s response. The experimenter held the dog for 4 seconds then said “Go!”, let the subject free to choose one of the two stimuli, which the dogs did by touching the monitor with the nose. The experimenter reopened her eyes as soon as the dog moved towards the monitors. If the dog chose the target stimulus, the experimenter gave verbal and food reward to the dog, then called it back into the starting position. If the dog chose the non-target stimulus, the experimenter called it back into the starting position without giving any reward.

#### Training phase

This phase was aimed at training dogs to discriminate a stimulus with a high percentage of coherently moving dots from a stimulus of randomly moving dots. Dogs underwent sessions of 20 consecutive trials, as described above. In each trial, the non-target (0% coherence) and the target stimulus (80% coherence) were presented. The side of presentation of the two stimuli was randomly chosen by the software and balanced within the 20 trials. Each dog underwent a maximum of 5 training sessions per day, with an interval between session of at least 20 minutes. Dogs were only fed at the end of the day, in the days in which they were involved in the study. Subjects could proceed to the subsequent test phase when they chose the target stimulus for at least 18 out of 20 trials (i.e. 90% accuracy) in 6 consecutive sessions, distributed over two separate days.

#### Test phase

This phase was meant to assess dogs’ threshold of perception of coherent motion. Sessions of this phase were composed of 24 trials. In the first 4 trials, dogs were presented with the same stimuli as those of the training phase (80% coherence), as a ‘warm-up’; another 10 of such training trials were randomly interposed with others among the rest of the session. Inclusion of these training trials in the test session aimed at maintaining dogs’ motivation and at further controlling the maintenance of subjects’ discriminative performance in the test phase. In the remaining 10 trials of each test session, test stimuli were presented, so that each level of coherence (i.e. 60%, 50%, 40%, 30%, and 20%) was presented twice within the session. Apart from the constraint that in the first 4 trials training stimuli were presented, and that the side of presentation was balanced for each type of stimulus, the order and side of presentation of training and test stimuli were randomized within each session. Each dog could complete a maximum amount of 5 test sessions per day with an interval between sessions of at least 20 minutes.

### Procedure for humans

The experiment was run in the same setting used for the dogs, with the exception that subjects sat on a stool at 150 cm from the monitors.

There was no preliminary training, but subjects received instructions on how to operate the keyboard, which they used to choose either the left or right monitor (by pressing left and right arrow keys, respectively). The timing of presentation of the stimuli was handled by the experimenter, who was sitting behind the subject. In order to expose the human subjects to the stimuli for the same amount of time as it was for the dogs, subjects could not choose before at least 4 s were elapsed from the appearance of the stimuli on the monitor. Once a subject had performed a choice, a black screen appeared for 5 s before the next presentation.

Human subjects underwent a training and a test phase similar to those described for dogs, with the only differences that in the training phase the learning criterion could be achieved within a single day and that in the test phase the maximum number of sessions that participants could complete within a single day was set at 10.

### Ethical statement

The experiment involving dogs did not cause any pain, suffering or distress; for the experiment on humans, participation was voluntary, the experiment did not involve any risk or distress, and all the information regarding the aim and the procedure of the experiment were given beforehand, and informed consent was obtained from all human participants. For these reasons, no need of approval by local Ethics Committee was required by our institutions, in accordance with the current European and Italian legislation.

### Data collection and statistical Analysis

Data about the choice performed by subjects in each trial were automatically collected with MATLAB (MATLAB version 7.10.0. Natick, Massachusetts: The MathWorks Inc., 2010) and, after the calculation of means. Data of each dog were fitted with a logistic function by using the routines provided by the Palamedes toolbox^35^, which considers a proportion of correct response for the level of coherence given by as:

$$P(C;\alpha ,\beta ,\gamma ,\lambda )=\gamma +\frac{1-\gamma -\lambda }{1+{e}^{-\beta (C-a)}}$$

As the task was a 2-alternative forced-choice, the lower asymptote for guess (*Gamma*) was set to 0.5, while the upper asymptote (*Lambda*) was fixed by setting the lapse rate to 0.02. The parameters *Alpha* and *Beta* were left free. *Alpha* refers to the threshold, i.e. the value along the abscissa corresponding to the coherence level at which the function attains its steepest point. *Beta* is a discrimination parameter often referred to as the “slope”.

An independents samples t-test was used to compare means of the *Alpha* and *Beta* parameters between our dogs and human participants.
